# Bill Length of Non‐breeding Shorebirds Influences the Water Depth Preferences for Foraging in the West Coast of India

**DOI:** 10.1002/ece3.70396

**Published:** 2024-10-22

**Authors:** K. M. Aarif, Jan Zouhar, Zuzana Musilova, Petr Musil, Aymen Nefla, Sabir Bin Muzaffar, K. A. Rubeena

**Affiliations:** ^1^ Department of Ecology, Faculty of Environmental Sciences Czech University of Life Sciences Prague Praha Suchdol Czechia; ^2^ Department of Biology, Faculty of Sciences of Tunis University of Tunis El Manar II Tunis Tunisia; ^3^ Department of Biology United Arab Emirates University Al Ain UAE; ^4^ Department of Science The Natural History Museum London UK; ^5^ Centre for Environment and Marine Studies, Research & Innovation King Fahd University of Petroleum & Minerals Dhahran Saudi Arabia

**Keywords:** bill size, foraging behaviour, shorebirds, sustainable conservation, water depth, wetlands

## Abstract

Body size, bill length and shape determine foraging techniques, habitat selection and diet among shorebirds. In this study, water depth preferences of different shorebirds with different bill sizes in various habitats including mudflats, mangroves at Kadalundi‐Vallikkunnu Community Reserve (KVCR) (19 shorebird species) and adjacent agroecosystems at Vazhakkad (12 species) were studied between 2017 and 2020. The bill length of the shorebirds was significantly and positively associated with the average water depth, where shorebirds were observed to forage. Shorebirds with shorter bill lengths preferred shallow waters and those with longer bills preferred deep waters for their foraging activities. Habitat type also had a significant effect on the shorebird occurrence. Eurasian Curlews in both mangroves and mudflats were observed in areas with a higher water depth compared to other species. This is due to the fact that shorebirds tend to specialise in feeding habitats or in prey items to reduce intraspecific competition and distribute themselves in space and time in accordance with the availability of their resources. The occurrence of some species in agroecosystems is attributed to the reduced food availability, habitat quality and other disturbances for shorebirds on tidal flats, which are critical for sustaining migratory phenology. The differences in bill morphology are crucial in determining diet, water depth, niche preferences and segregation. Morphological characters and hydrological rhythms determine specialisation in diet and habitat preference in shorebirds.

## Graphical abstract

Body size, bill length and shape determine the foraging techniques, habitat selection and diet among shorebirds. In this study, water depth preferences of different shorebirds with different bill sizes in various habitats were investigated between 2017 and 2020. Average water depth, where shorebirds were observed, was significantly and positively associated with their bill lengths. This study underscores the need for conservation of wetlands with its diverse hydrological rhythms to preserve the biodiversity.

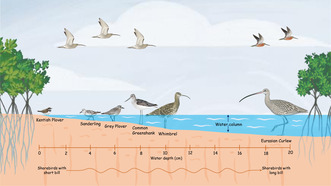

## 1 Introduction

Aquatic birds typically depend on wetlands and coastal shorelines for foraging, loafing and moulting (Lee, Jabłoński, and Higuchi 2007; Muñoz‐Pedreros and Merino 2014; Debela et al. 2020). Shorebirds (Charadriidae) are a diverse group of aquatic birds, which can be sampled on a large spatial scale using sophisticated sampling techniques compared to most other animals (Lu et al. 2022). Shorebirds utilise intertidal wetlands, agroecosystems, salt pans and sand beaches as their foraging grounds (Elphick, Shriver, and Greenberg 2022). Aquatic birds including shorebirds and water birds are key components of intertidal wetlands and coastal ecosystems.

Body size, bill length and shape have a major role in their foraging behaviour (Yu et al. 2023), selecting microhabitat for foraging (Zhang et al. 2014) and diet preference (Zhang et al. 2019; Probst, Ralston, and Bentley 2022; Xu et al. 2023). The foraging manoeuvres of shorebirds with long bills include probing the deep sediments and plunging or sweeping bill in the water, whereas shorebirds with short bills perform routing and pecking manoeuvres at the substrate/sediment surface (Angarita‐Báez and Carlos 2023). The shorebirds with shorter bill length tend to avoid foraging at tidal flats during day time, since the profitable prey items such as polychaetes may be dwelling deep inside due to high sediment temperature and are unreachable to the short‐billed birds (Linhart et al. 2022). The foraging depths are influenced by culmen and tarsus lengths also (Linhart et al. 2022).

Bill shape (straight or curved) influences the foraging techniques used by many shorebirds (Angarita‐Báez and Carlos 2023). Straight bills help in pecking of epifaunal invertebrates, while the curved bills are associated with probing of infaunal prey (Mishra, Kumar, and Kumar 2019). Efficiency of the tactile foraging strategy is determined by the penetration capacity of the bill, which in turn is influenced by its curvature and length (Mishra, Kumar, and Kumar 2019). Generally, the ideal morphological features for a bill are to be long and narrow, with the anterior portion flattened to facilitate penetration (Moermond 1990). In addition, the foraging time/duration varies with respect to the size of the bird (Zwarts, Bijlsma, and van der Kamp 2023). Larger birds usually use less foraging time than smaller birds, as they are capable of feeding larger beneficial prey within less time duration (Zwarts, Bijlsma, and van der Kamp 2023). These reports underscore the need for investigating the relationship between foraging depths and strategies and the bill length and shape of the shorebirds.

Water depth is one of the three important ecological variables that determine the suitability of a habitat for shorebirds, type of prey items and vegetation being the other two (Sorensen, Hoven, and Neill 2020). Different shorebird species exhibit different niche preferences based on these variables. Habitat preference by shorebirds is driven by the habitat quality along with the water depth preference and bill length (Collazo, O'Harra, and Kelly 2002; Strum et al. 2013; Schaffer‐Smith et al. 2018).

Common Greenshanks (*Tringa nebularia*) and Spotted Redshanks (*T. erythropus*) with similar morphological characteristics use similar microhabitats and foraging techniques (Yu et al. 2023). Common Greenshanks often forage at shallow water areas in the wintering grounds and forage deeper into the mud near shallow water (Yu et al. 2023). Shallow, wetland areas of 0.1–9.2 cm water depth attract shorebirds (Philippe et al. 2016). Moreover, sustainable conservation of deeper waters could increase overall environmental heterogeneity needed for common coots and supported some larger‐sized wading bird species (Yijin, Mingqin, and Quanjiang 2020).

Leg length (tarsus) is positively correlated with the water depth, and the shorebirds with short legs tend to forage in shallow water areas (Norazlimi and Ramli 2015). Many shorebirds prefer their foraging water depths to be < 10 cm (Elphick and Oring 1998; Strum et al. 2013; Schaffer‐Smith et al. 2018), whereas smaller shorebirds such as sandpipers (*Calidris sp*.) with short bills and short legs have the narrowest habitat specifications, foraging in the flooded wetlands of < 5 cm depth (Collazo, O'Harra, and Kelly 2002; Taft et al. 2002; Ma et al. 2010). Prey tend to concentrate at shallow water areas and facilitate the foraging efficiency of small shorebirds (Neckles, Murkin, and Cooper 1990; Ma et al. 2010; Schaffer‐Smith et al. 2018). However, shallow water areas are confronting a massive degradation globally and a study reported the loss of shallow water habitats in the Central Valley of California during spring and fall migration (Barbaree et al. 2015; Dybala et al. 2017).

Habitat preferences of different shorebirds with respect to bill and leg length and water depth preferences at Indian coasts are yet to be explored. Kadalundi‐Vallikkunnu Community Reserve (KVCR) coastal wetlands in the west coast of India are known to host internationally important numbers of migratory waterbirds in the Central Asian Flyway (Aarif et al. 2014, 2020, 2021; Rashiba et al. 2022). The inland agroecosystems also provide alternate support and easy prey accessibility to the shorebirds (Byju et al. 2024).

Variations in water depth of the habitats influence the species composition and abundance of wetland birds (Baschuk et al. 2012). Water depth preferences of non‐breeding shorebirds foraging or wintering at KVCR are not well characterised. Spatial and temporal patterns of water depth distributions and knowledge on the water depth preferences by the shorebirds can assist in more targeted sustainable strategies to optimise habitat quality and quantity across wetlands managed by government authorities. Water depth distribution is crucial in determining the quality and quantity of habitat for foraging shorebirds (Lyons et al. 2008). Water depth distributions at key wintering and foraging grounds of shorebirds and the factors that determine the water depth preferences of shorebirds are yet to be explored, and it is necessary to map optimal shallow water habitats accessible to both smaller and larger shorebirds during migration. The objectives of this study were to determine the effects of bill length and tarsus length on foraging water depth preference in selected species of shorebirds in different coastal habitat types in the west coast India.

## 2 Materials and Methods

### 2.1 Study Area

**FIGURE 1:**
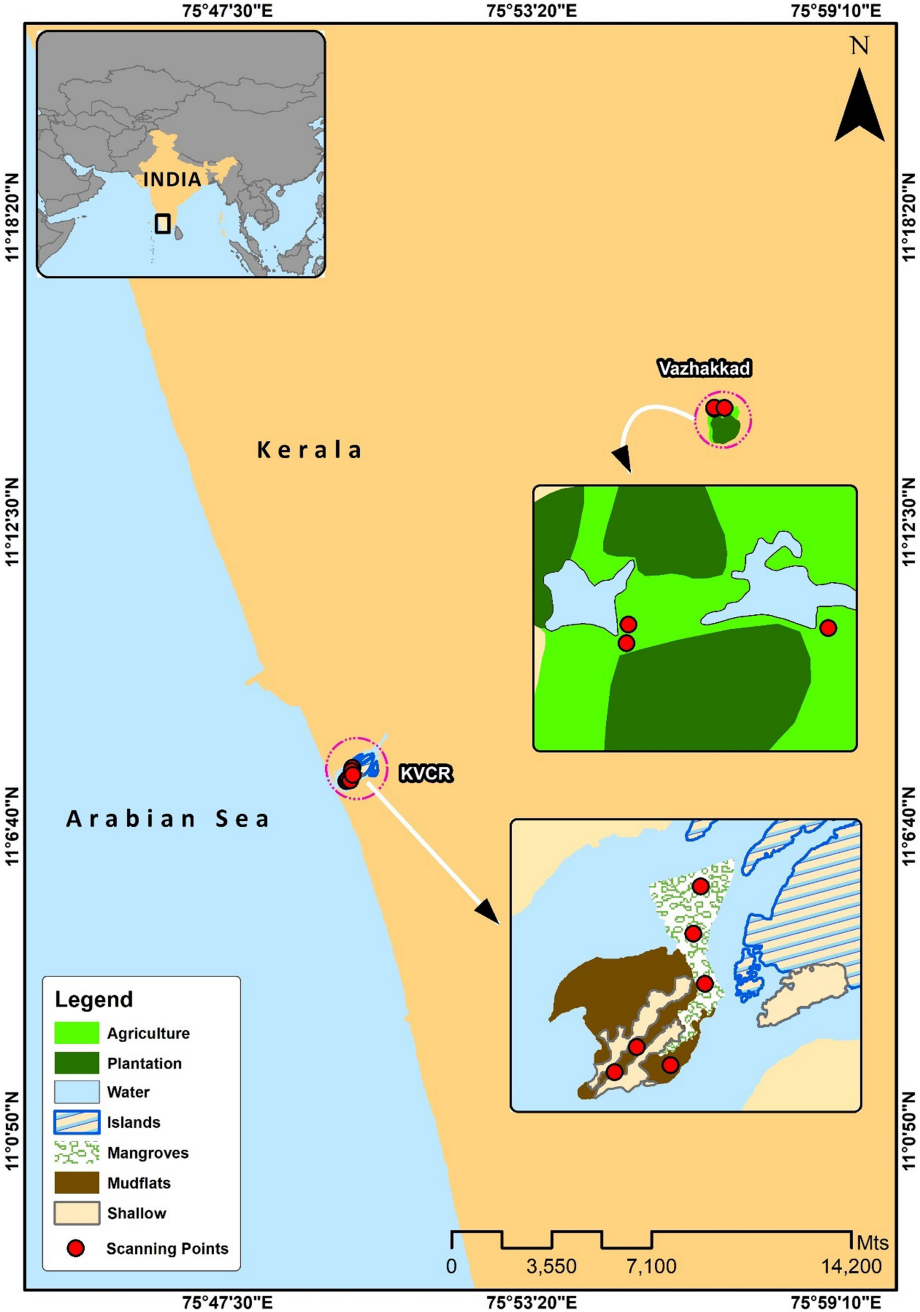
Map of study area at the Kadalundi‐Vallikkunnu Community Reserve (KVCR) and Vazhakkad in Kerala, southwestern India. Three scanning points in each site are selected to study the water depth preferences of shorebirds.

The KVCR is located in the area where the Kadalundi River drains into the Arabian Sea on the West Coast of Kerala (Figure 1). At the river mouth, the Kadalundi River divides into two parts around a small island between them. There are patches of 8 ha of mangroves (11°07′43.2″ N 75°49′50.7″ E) and 8 ha of mudflats (11°07′37.5″ N 75°49′48.6″ E), which forms the two major habitats for the wintering shorebirds in the KVCR wetland. Mudflats are exposed during low tides and offer potential foraging grounds for several hundreds of wintering non‐breeding shorebirds (Aarif, Prasadan, and Babu 2011; Aarif and Mussammilu 2018; Aarif et al. 2021).

The Vazhakkad agroecosystem is situated on the banks of the river Chaliyar (11°14′36.9″ N 75°57′03.0″ E), which spans an area of approximately 37.06 ha. The fallow period is from April to October; the cultivation includes paddy (October to April) and banana as crop rotation. Subsequently, the field dries, and with the onset of monsoon, grasses and other small plants grow. Agricultural land hosts orthopterans, frogs and fishes during the monsoons. During the post‐monsoon season (October, November, December and January), the majority of this wetland is in a waterlogged condition, which leaves a thin layer of water to be retained until the onset of summer (February, March, April and May) (Byju et al. 2024).

## 3 Dataset Structure and Variables

### 3.1 Shorebird Sampling

The survey was carried out from January 2017 to December 2020, at the mudflats and mangroves of KVCR, specifically during low tide. This study was conducted once a week (6.00–11.00 am) in the months of January, February, March, April, May, September, October, November and December. The survey of Vazhakkad agroecosystems was undertaken once a week, between 7.30 and 11.30 am, exclusively during November, December and January, spanning the years 2017–2020. This timing was selected due to its alignment with the optimal conditions for the presence of shorebirds.

We deployed three sets of video cameras (two Nikon Coolpix p1000 and a Nikon D500 with Nikon 200–500 mm telephoto lens) at three permanent scanning points covering the study area (mudflats, mangroves and Vazhakkad agroecosystem), with the help of two field assistants, to record the water depth preferences of shorebirds during the study period (Figure 1). The videos were recorded continuously during the study period from 7.00 to 11.30 by ensuring enough battery backup for the same. After recording their behaviours, all video footages were transferred to the software and analysed by the play‐replay method (Kuwae 2007). Briefly, in the play‐replay method, we analysed all video footages by replaying all observations of birds at different depths.

A total of 19 species of shorebirds were selected from the KVCR, and 12 species were chosen from the Vazhakkad agroecosystem to study water depth preferences (Table S1). Lesser Sand Plover (*Charadrius mongolus*), Greater Sand Plover (*Charadrius leschenaultii*), Kentish Plover (*Charadrius alexandrinus*), Pacific Golden Plover (*Pluvialis fulva*), Grey Plover (*Pluvialis squatarola*), Common Sandpiper (*Actitis hypoleucos*), Terek Sandpiper (*Xenus cinereus*), Marsh Sandpiper (*Tringa stagnatilis*), Sanderling (*Calidris alba*), Little Stint (*Calidris minuta*), Dunlin (*Calidris alpina*), Curlew Sandpiper (*Calidris ferruginea*), Eurasian Oystercatcher (*Haematopus ostralegus*), Black‐winged Stilt (*Himantopus himantopus*), Common Redshank (*Tringa totanus*), Common Greenshank (*Tringa nebularia*), Bar‐tailed Godwit (*Limosa lapponica*), Whimbrel (*Numenius phaeopus*) and Eurasian Curlew (*Numenius arquata*) were recorded in different water depth preferences from mudflats and mangroves of KVCR. However, Little Ringed Plover (*Charadrius dubius*), Pacific Golden Plover, Grey Plover, Marsh Sandpiper, Grey headed Lapwing (*Vanellus cinereus*), Green Sandpiper (*Tringa ochropus*), Wood Sandpiper (*Tringa glareola*), Red wattled Lapwing (*Vanellus indicus*), Common Sandpiper, Common Snipe (*Gallinago gallinago*), Black‐winged Stilt and Common Greenshank were documented in different water level preferences from the agroecosystem of Vazhakkad (Table S1).

### 3.2 Water Depth Monitoring

**FIGURE 2:**
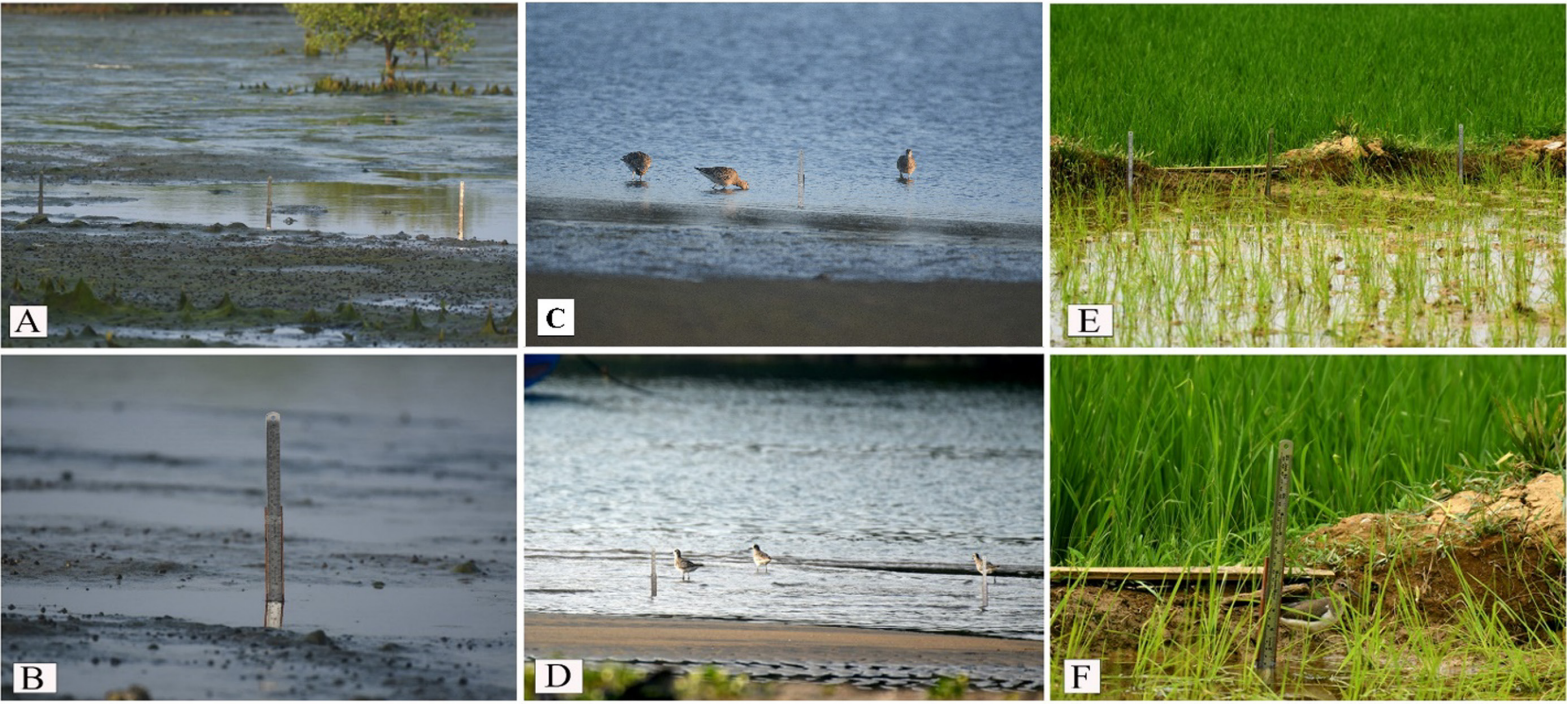
The study area showing the setup consisting of a graduated ruler permanently positioned at different locations to indicate water depth preferences of shorebirds at (A) mangroves and (B–D) mudflats in KVCR and (E, F) the Vazhakkad agroecosystem.

Water depth is the depth of the water column (cm) at which each shorebird species was observed to forage across the three habitats. The depth was measured using long measuring scales set up permanently at several locations (Figure 2). Concretely, to account for habitat‐specific variation in water depth preferences, we chose 10 locations at random across each habitat type to measure water depth. In the play‐replay analysis of the video footage, the water depth observations were recorded as the average water depth at which a group of individuals (5.13 individuals on average) of a concrete species were foraging for a continuous time period. Throughout the study period, a total of 1995 observations were documented in 113 monitoring days, with 697 from mudflats, 370 from mangroves and 928 from agricultural lands.

The shorebird species at mudflats and mangroves were classified into seven groups and that of Vazhakkad agroecosystem into five groups according to their water depth preferences (Table S1).

Additionally, we used data on bill and tarsus length of shorebirds documented in the literature (Lee and Hockey 2001; Nebel 2005; Nebel and Thompson 2011; Lank et al. 2017) for further statistical analysis to establish the relationships of various bill length and water depth preferences at mudflats, mangroves and agroecosystem.

### 3.3 Statistical Analysis

The dependent variable in our analyses was the average *water depth*, measured for a group of individuals (of a recorded group size) during a monitoring session. Continuous explanatory variables included bill length and tarsus length. Moreover, we included categorical variables indicating the species, year, month and habitat.

We ran a mixed‐effects linear regression explaining average water depth, with random effects at the level of species (to avoid pseudo replication), with cluster‐robust standard errors (clustered at the level of species). The continuous data was log‐transformed to remove skewness, and then the following models were considered: (i) continuous variables water depth and tarsus length; (ii) models with and without month as a covariate and (iii) models either unweighted (1 group = 1 observation) or with observations weighted by the group size.

The regression analyses have been performed in Stata 18 (StataCorp 2023). The Stata code that reproduces our results is included online as Supporting Information. To provide a means of reproducing our results using open‐source software, we also provide an R code that performs the regressions; however, we could not find a straightforward way to obtain cluster‐robust standard errors in R for mixed‐effects regressions, meaning that the results regarding statistical inference differ to some extent.

## 4 Results

The regression results in Tables 1 and 2 tell a consistent story, regardless of the different weighting schemes: the bill length was, in all cases, a statistically and practically significant predictor of water depth preference. The coefficients on bill length imply that a 1 mm increase in bill lengths is associated with a similar increase (0.099 cm in Table 1, 0.096 cm in Table 2) in average water depth. It can be noted that the coefficient on tarsus length is both much smaller in magnitude and statistically insignificant.

**TABLE 1:** Results of mixed‐effects linear regression explaining log‐transformed and original (level‐form) average water depth (unweighted models with and without month as a covariate).

	Log of water depth			
	Coefficient	*p*	Coefficient	*p*
Log of tarsus	0.0528	(0.833)	0.0403	(0.872)
Log of bill	1.073***	(0.000)	1.079***	(0.000)
Year				
2017	Ref.		Ref.	
2018	−0.00287	(0.928)	−0.000123	(0.995)
2019	0.00197	(0.942)	−0.00107	(0.962)
2020	−0.0642	(0.560)	−0.0494	(0.535)
Habitat				
Agroecosystem	Ref.		Ref.	
Mangroves	0.445	(0.248)	0.524	(0.221)
Mudflats	0.526	(0.162)	0.618	(0.142)
Month				
Jan			Ref.	
Feb			−0.0230	(0.810)
Mar			−0.0205	(0.834)
Apr			−0.0680	(0.587)
May			−0.0739	(0.363)
Sep			−0.0401	(0.684)
Oct			0.0944	(0.330)
Nov			0.109	(0.705)
Dec			0.0761	(0.073)
Constant	−3.112***	(0.000)	−3.189***	(0.000)
Log of *σ*(species)	−0.859***	(0.000)***	−0.868***	(0.000)
Log of *σ*(residual)	−0.547***	(0.000)	−0.550***	(0.000)
Observations	1929		1929	

*Note:* *p*‐values reported in parentheses: ***p < 0.001.

Abbreviations: Ref. = reference category, *σ*(species) and *σ*(residual) = standard deviation of the species random effect and the idiosyncratic random error, respectively.

**TABLE 2:** Results of mixed‐effects linear regression explaining log‐transformed and original (level‐form) average water depth (models weighted by the number of shorebirds with and without month as a covariate).

	Log of water depth			
	Coefficient	*p*	Coefficient	*p*
Log of tarsus	0.234	(0.396)	0.208	(0.446)
Log of bill	0.909***	(0.000)	0.926***	(0.000)
Year				
2017	Ref.		Ref.	
2018	0.0311	(0.485)	0.0296	(0.247)
2019	0.0344	(0.482)	0.0335	(0.439)
2020	0.0110	(0.881)	0.0227	(0.679)
Habitat				
Agroecosystem	Ref.		Ref.	
Mangroves	0.804*	(0.025)	0.851*	(0.032)
Mudflats	0.949**	(0.007)	1.001*	(0.011)
Month				
Jan			Ref.	
Feb			−0.0168	(0.722)
Mar			0.00836	(0.881)
Apr			0.0564	(0.292)
May			−0.0767	(0.179)
Sep			−0.0324	(0.541)
Oct			0.0791	(0.091)
Nov			0.110	(0.675)
Dec			0.0894**	(0.001)
Constant	−3.514***	(0.000)	−3.566***	(0.000)
Log of *σ*(species)	−0.809***	(0.000)	−0.807***	(0.000)
Log of *σ*(residual)	−0.875***	(0.000)	−0.881***	(0.000)
Observations	1929		1929	

*Note:* *p*‐values reported in parentheses: *p < 0.05, **p < 0.01, ***p < 0.001.

Abbreviations: Ref. = reference category, *σ*(species) and *σ*(residual) = standard deviation of the species random effect and the idiosyncratic random error, respectively.

**FIGURE 3:**
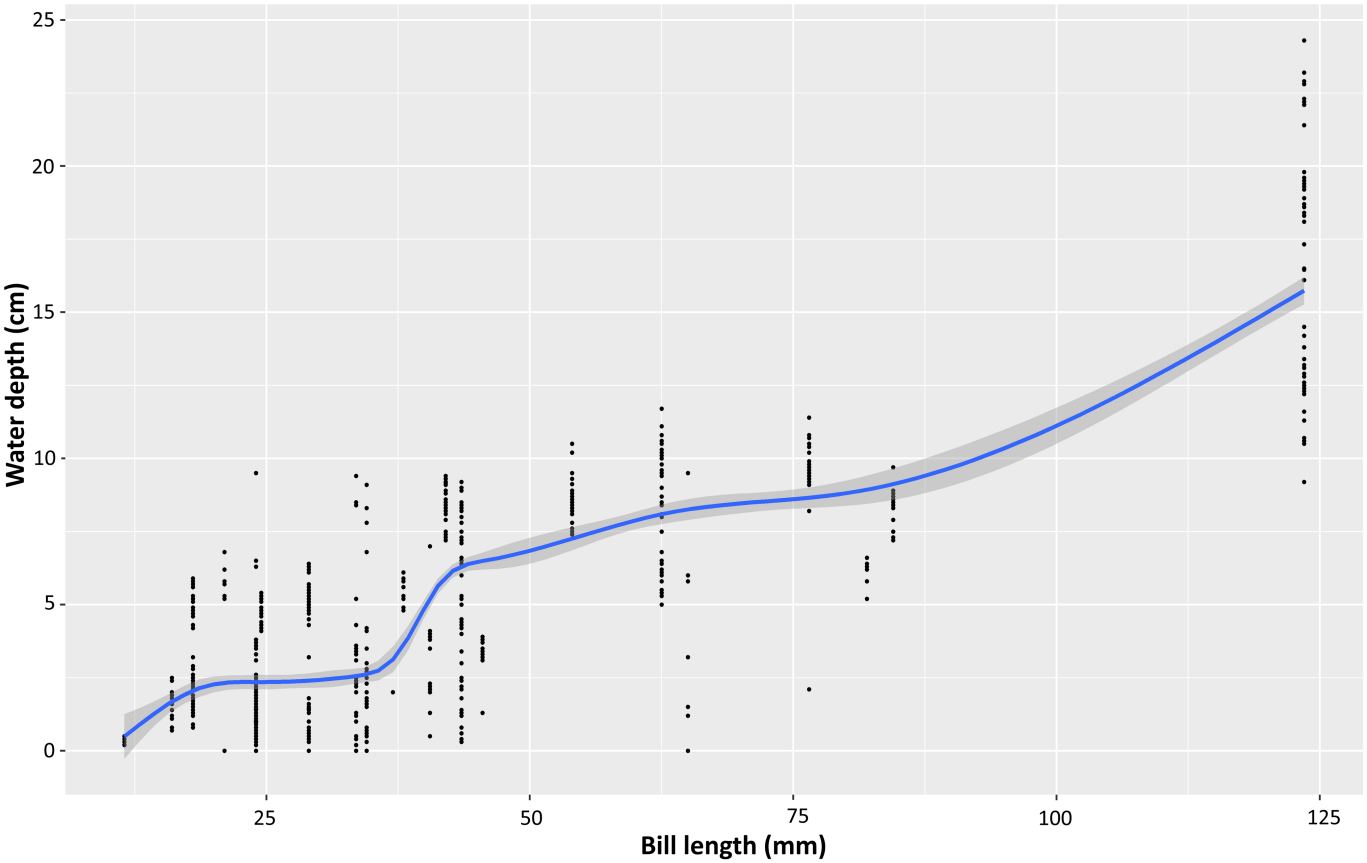
Relationship between preferred water depth and bill length of the selected non‐breeding shorebirds at mudflats and mangroves in KVCR and Vazhakkad agroecosystem during the study period (scatterplot smoothing was done using LOESS; points have been jittered horizontally to enhance readability of the plot).

**FIGURE 4:**
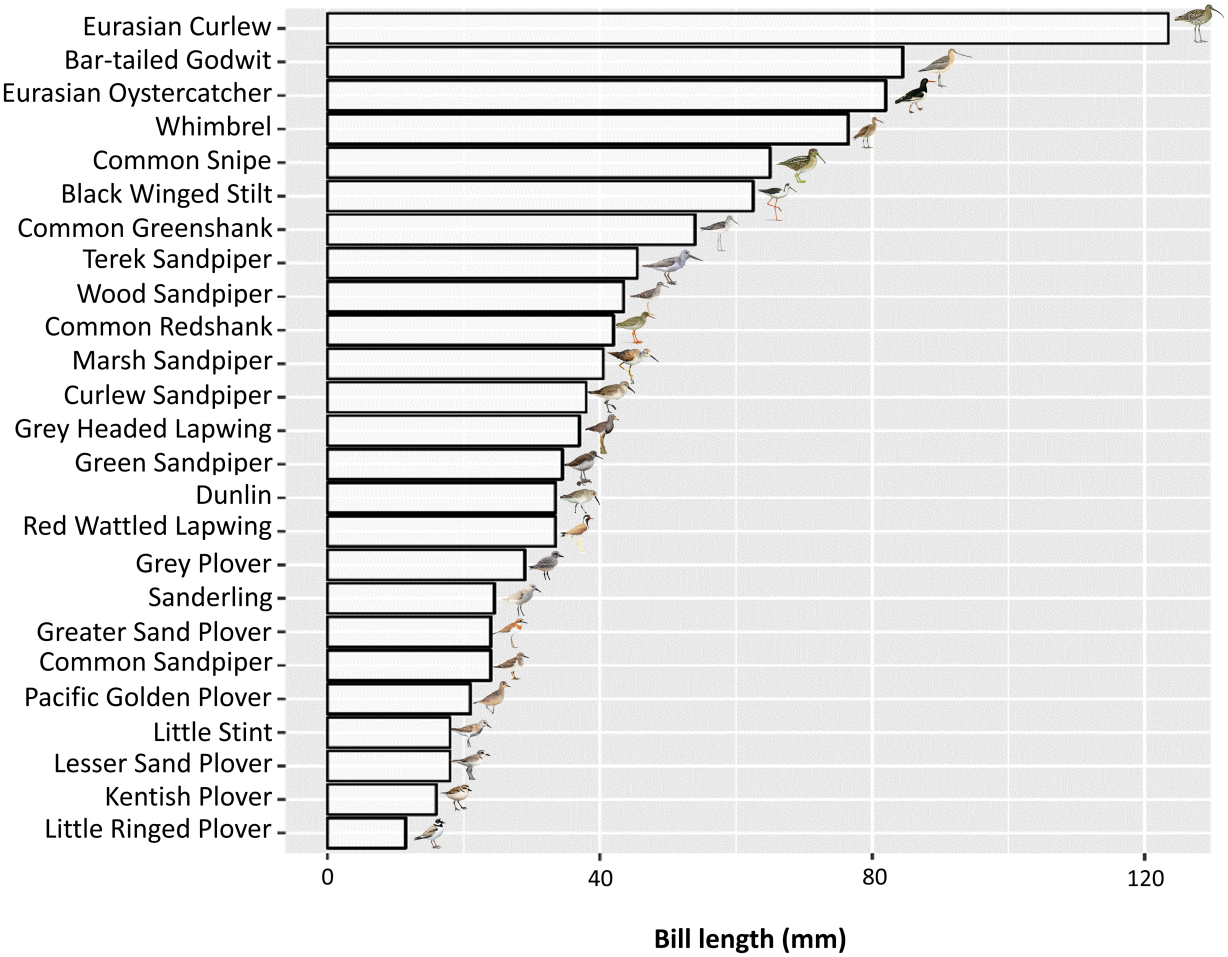
Comparative bill lengths (in mm) of selected non‐breeding shorebirds.

Figure 3 visualises the relationship between the bill length and the average water depth using non‐parametric locally estimated scatterplot smoothing (LOESS). The pileups of observations at concrete bill length values correspond to the average bill lengths of individual species. Eurasian Curlew (*Numenius arquata*) was the species with the longest bill among the studied species (90–175 mm), while Little Ringed Plover (*Charadrius dubius*) (12.8 mm) had the shortest bill among the species (Zhao 2001; Summers et al. 2013; Yu et al. 2023). A graphical overview of bill lengths is available in Figure 4.

**FIGURE 5:**
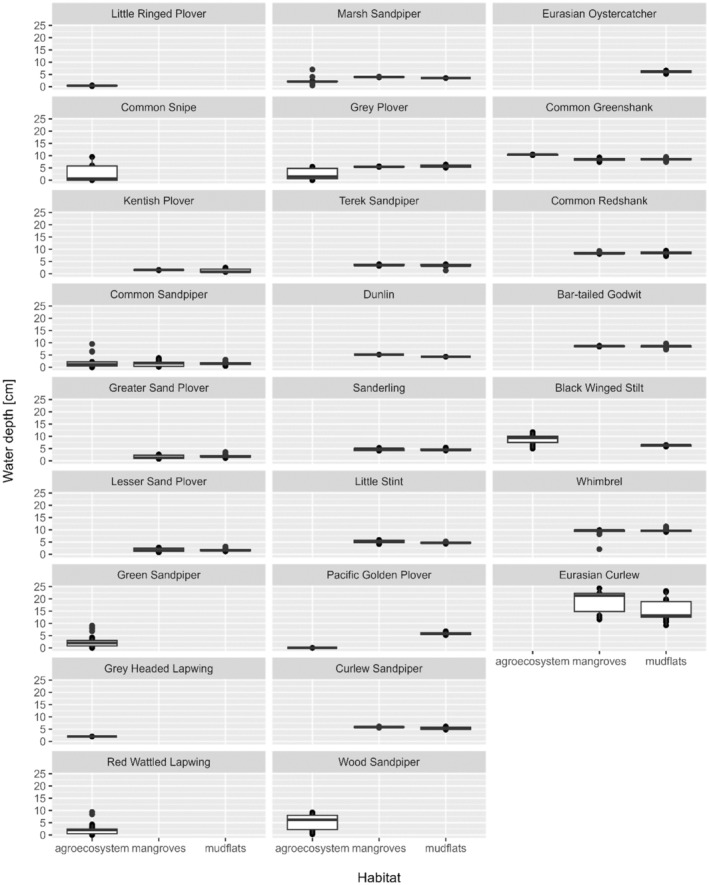
Water depth preferred by each shorebird species from all three habitats—agroecosystem, mangroves and mudflats in Kadalundi_Vallikunnu Community Reserve and Vazhakkad agroecosystem during the study period. Species are ordered column‐wise according to the median water depth across all habitats.

Table 2 shows that the type of habitat has a significant effect on average water depth. However, some individual species were observed in deeper water mudflats and shallower water agriculture land (e.g., Grey Plover and Pacific Golden Plover) (Figure 5). Also, species specialised in certain habitat types for feeding, and some other species are observed in agroecosystems. Eurasian Curlews in both natural habitat types (i.e., mangroves and mudflats) were observed in areas with a higher water depth compared to other species.

## 5 Discussion

Shorebird occurrence was significantly associated with the type of habitat. Shorebirds tend to specialise in feeding habitats or in prey items to reduce intraspecific competition and distribute themselves in space and time in accordance with the availability of their resources (Jourdan et al. 2021). We demonstrate that water depth preference of shorebirds is influenced by their bill length. In addition, the bill length also influences their feeding strategies and specialisation of feeding habitats. This substantiates the possible association of the differential spatial distribution of the shorebirds on mudflats, mangroves and agricultural lands and their specialisation on certain microhabitats or on particular species of invertebrates in their diet. Previously, Xu et al. (2023) reported that the diet preferences and habitat density are important predictors of variations in bill length and tarsus length among non‐migratory birds. Also, shorebirds with shorter legs tend to specialise in shallow areas (Elphick and Oring 1998). In terms of non‐breeding habitat use, the coastal specialist shorebird community may be more limited in their use of non‐tidal habitats (Jackson et al. 2021). The presence of some species in agroecosystems could be attributed to the reduced food availability, habitat quality and other disturbances for shorebirds on tidal flats, which are apparently critical for maintaining their regular migration (Byju et al. 2024). In order to maintain the high prey capture rate, the short‐billed birds tend to relocate themselves to habitats where there is sufficient prey availability (Deboelpaep et al. 2023). Morphological characters, particularly bill length and shape and tarsus length of shorebirds, influence their habitat preferences and foraging strategies, which in turn determines the dietary specialisation (Trevail et al. 2021; Jourdan et al. 2021).

**FIGURE 6:**
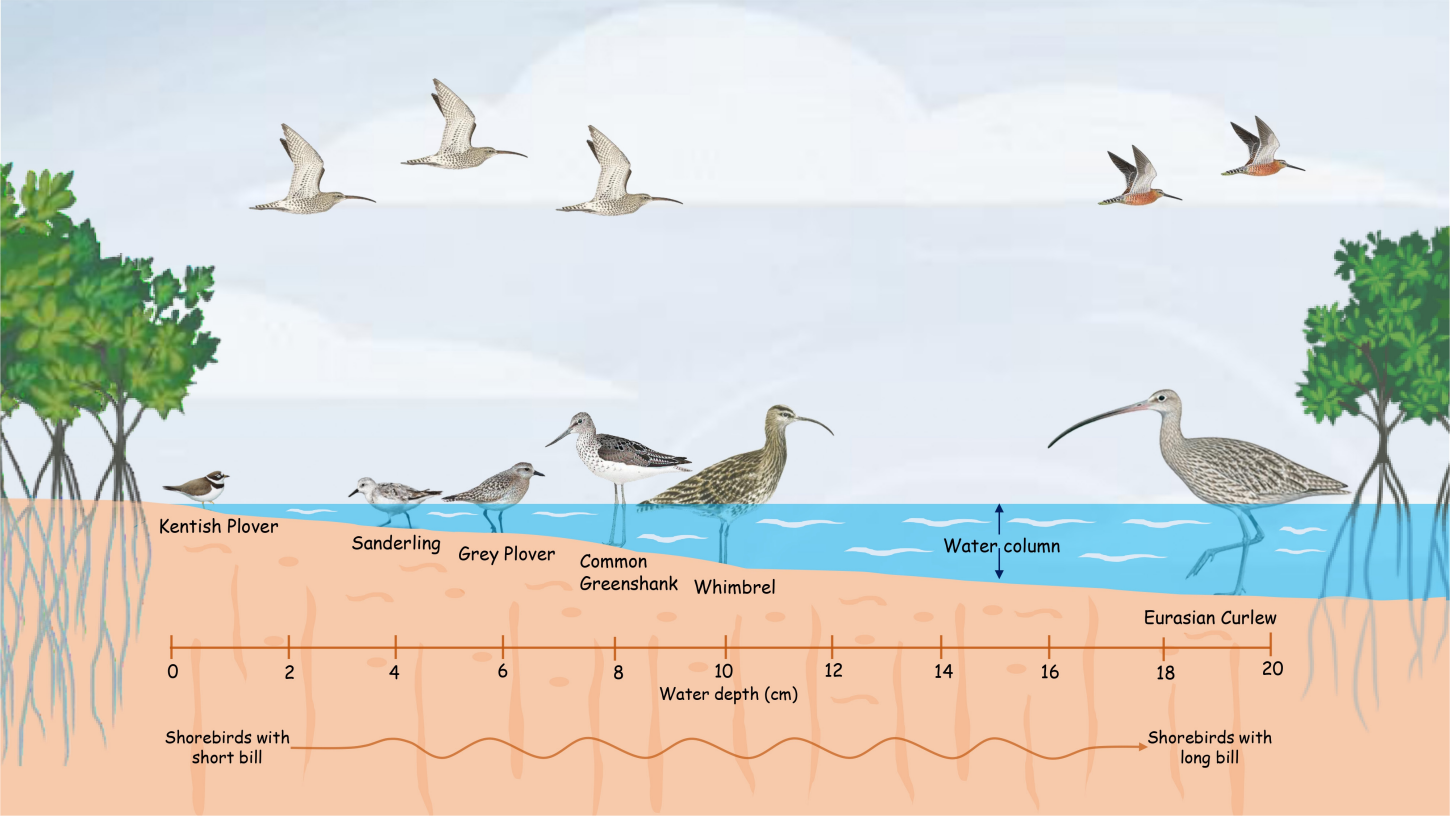
A pictorial representation of water depth preference among various shorebird species with different bill and leg lengths. The water depth preference of shorebirds is associated with their bill length.

The ability to exploit a particular prey requires knowledge of its distribution and a way to access them (Bolnick et al. 2003). Any minute variations in the bird's morphological characteristics could influence its foraging strategies (Moermond 1990). Foraging time, prey size, probing depth and habitat preference are all influenced by variations in the size of the bill and the length of the legs in shorebird species (Norazlimi and Ramli 2015). We found a significant positive association between shorebirds’ choice of water depth for foraging and their bill lengths. This is consistent with findings of Norazlimi and Ramli (2015), who stated that longer bills indicated deeper foraging, larger prey and longer foraging periods. The differences in bill morphology are crucial in determining diet, water depth and niche preferences and segregation (Franks et al. 2013). Foraging specialisation studies on Black‐tailed Godwits suggest that there are intraspecific differences in specialisation in diet and habitat use, females being more specialist feeders than males (Catry et al. 2014). Thus, the individual differences in specialisation can be attributed to the sexual dimorphism in bill length. Similarly, the female adult Western Sandpipers exhibited dietary preference towards polychaetes with increase in bill length (Hall et al. 2021). According to our observations, Eurasian Curlews with the longest bills and tarsus were found in areas with higher water depths in both mangroves and mudflats than other species (Figure 6). Longer bills facilitate effective probing in sites with more water content (Aung et al. 2022).

Our findings substantiate that the species with shorter bills forage in shallow water using pecking and probing techniques. However, certain large‐sized species, like the Grey Plover and Pacific Golden Plover, have been seen in both deeper water mudflats and shallow agricultural land. Generally, plovers are well‐known for their visual foraging methods, which include a run‐stop‐search strategy and pecking to pierce the surface of a substrate (Angarita‐Báez and Carlos 2023). Lesser Sand Plovers have been found to prefer consuming crustaceans employing the run‐stop‐chase strategy in the KVCR habitat. However, similar to our finding that the large species prefer deep waters, Masero, Estrella, and Sánchez‐Guzmán (2007) observed that certain large species such as Grey Plovers, forage consistently on pelagic prey items in water column during high tides, just as the foraging strategy of sandpipers. It was opined that feeding at high tide was an opportunistic foraging technique used by shorebirds, when they fail to acquire enough feed during low tides, to meet the high energy requirement associated with the long‐distance migration (Masero 2002). The larger nereidid polychaetes, similar to those eaten by Common Redshank and Eurasian Curlews (Goss‐Custard et al. 2006), were the main prey items identified as being consumed by Grey Plovers and Pacific Golden Plovers (Iwamatsu, Suzuki, and Sato 2007; Kato, Omori, and Yoneda 2000). Thus, the large‐sized plovers in our study area may be forced to choose to consume pelagic prey due to this interspecific competition caused by overlapping niches and their long bills might have assisted them in this foraging technique. This small‐prey profitability enables them to consume prey types that swim freely in the water without any fear of predatory birds (Estrella, Masero, and Pérez‐Hurtado 2007). The diversity and abundance of invertebrates on the same tidal flats determine the stability of those areas as a significant source of food for migratory shorebirds (Sato and Nakashima 2003). The rate of polychaetes intake by shorebirds indicates their abundance, availability and accessibility of particular species in the sediment. Aarif et al. (2021) found a marked decrease in the amount of invertebrate prey items in the intertidal habitats of the study area, which in turn results in an escalated competition among species for the remaining resources. The species' attempt to become less vulnerable to environmental changes can be seen in the individual variation observed in the current study.

The habitat degradation caused by the alterations in the sediment properties over time (Rubeena et al. 2023) and heavy metal (Aarif et al. 2023) and microplastics pollution (Athira et al. 2024) due to anthropogenic impacts contributed significantly towards sediment hardening (Aarif et al. 2021) and resultant prey depletion (Rubeena et al. 2023). In a study conducted at 11 tidal flats in Australia, the biomass and intake of Eastern Curlew were negatively correlated with the substrate penetrability (Finn and Catterall 2023). Due to the depth, substrate hardness or prey behaviour, it may be more challenging for shorebirds to extract polychaetes in some habitats (Finn, Catterall, and Driscoll 2008; Duijns et al. 2014), which would necessitate increased efforts to achieve adequate intake rates to meet their energy needs. Thus, the increased reliance of migratory shorebirds on smaller pelagic prey items may also be a result of the sediment hardening observed in the KVCR mudflats (Rubeena et al. 2023). KVCR, being an important wintering site for the migratory shorebirds, serves as a nutritional landscape (Rubeena et al. 2023) to meet their high energy demands for long‐distance migration. Sediment hardening, heavy metal pollution and other human impacts caused a decline in sediment penetrability, prey abundance and availability, making it tough and expensive for the migratory shorebirds to meet their nutritional requirements (Aarif et al. 2021). Considering all these factors, short‐billed migratory birds may find it more difficult to reach out to its prey. This was followed by switching of foraging activities of these migratory shorebirds to adjacent sand beaches (Aarif et al. 2014) and agricultural lands (Byju et al. 2024). Habitat heterogeneity, seasonal environmental variations and temporal variations in prey availability influenced their foraging ecology and trophic plasticity (Gliesch et al. 2023). However, this habitat switching is expensive in terms of energy expenditure and diversity of prey items (VanDusen, Fegley, and Peterson 2012). Putting all these together, it is clear that immediate actions by policy makers and stakeholders to restore the ecosystem health is imperative for the conservation of the habitats and its biodiversity. Sustainable measures are warranted to conserve the natural habitats as well as the agroecosystems to preserve the biodiversity and the abundance of micro and macro benthos and migratory shorebirds. This could include management through habitat protection measures, restoration and remediation of degraded wetlands, maintenance of adequate vegetation and water levels and education and outreach programs.

## 6 Conclusion

The study substantiates that the bill length is positively associated with the water depth preferences of foraging shorebirds. In other words, the foraging shorebirds are distributed in the natural habitats in accordance with their bill length, which in turn determines their foraging techniques. The current study emphasises the importance and influence of morphological characters and hydrological rhythms in the specialisation of diet and habitat preferences of shorebirds. Conservation of habitats and restoration of ecosystem health is essential in this perspective. Further investigations on the influence of sexual dimorphism and age structure in bill length, individual foraging skills or diet choices and other methods involved in the specialisation in niche and diet preferences are much needed to study the mechanisms underlying the prevalence of shorebirds at certain microhabitats and resource partitioning among them. Furthermore, the study warrants the need for implementing sustainable conservation measures to preserve the natural habitats such as mudflats, mangroves and agroecosystems as the ecosystem health impacts the foraging non‐breeding shorebirds.

## Author Contributions

**K.M. Aarif:** conceptualization (equal), data curation (equal), formal analysis (equal), methodology (equal), validation (equal), writing – original draft (equal), writing – review and editing (equal). **Jan Zouhar:** formal analysis (equal), writing – original draft (equal), writing – review and editing (equal). **Zuzana Musilova:** formal analysis (equal), validation (equal), visualization (equal), writing – review and editing (equal). **Petr Musil:** writing – original draft (equal), writing – review and editing (equal). **Aymen Nefla:** software (equal), writing – original draft (equal), writing – review and editing (equal). **Sabir Bin Muzaffar:** supervision (equal), validation (equal), visualization (equal). **K.A. Rubeena:** supervision (equal), validation (equal), visualization (equal), writing – original draft (equal), writing – review and editing (equal).

## Conflicts of Interest

The authors declare no conflicts of interest.

## Supporting information


**Table S1.** The list of shorebirds observed at different habitats and their grouping according to their water depth preferences.

## Data Availability

The data that support the findings of this study are openly available at: Aarif, K. M. and Muzaffar, Sabir Bin 2024. “Bill Morphology Influences the Water Depth Preferences by Winter Migrant Shorebirds in Critical Stopover Sites along the West Coast of India.” Mendeley Data, V1. doi: 10.17632/f2vpbpkgx9.1.
