# Supplementary material for: Bill Length of Non‐breeding Shorebirds Influences the Water Depth Preferences for Foraging in the West Coast of India
Source: Ecol Evol. 2024 Oct 22;14(10):e70396. doi: 10.1002/ece3.70396 (PMC11494249; doi:10.1002/ece3.70396)
Supplement: Supplementary file 1 — Table S1. The list of shorebirds observed at different habitats and their grouping according to their water depth preferences. [file ECE3-14-e70396-s001.docx]

**Supplementary Table 1:** The list of shorebirds observed at different habitats and their grouping according to their water depth preferences.

| Group | Water depth preference (cm) | Shorebird species |
| --- | --- | --- |
| Mudflats and Mangroves | | |
| I | 0.7 to 2.5 | Lesser Sand Plover, Greater Sand Plover, Kentish Plover |
| II | 3 to 4 | Common Sandpiper, Terek Sandpiper, Marsh Sandpiper |
| III | 4 to 5 | Sanderling, Little Stint, Dunlin, Curlew Sandpiper |
| IV | 5 to 6 | Pacific Golden Plover, Grey Plover, Eurasian Oystercatcher, Black-winged Stilt |
| V | 8 to 9 | Common Redshank, Common Greenshank, Bar-tailed Godwit |
| VI | 9 to 10 | Whimbrel |
| VII | 17 to 22 | Eurasian Curlew |
| Vazhakkad Agroecosystem | | |
| I | 0 to 1 | Pacific Golden Plover, Little Ringed Plover |
| II | 0 to 7 | Grey Plover, Marsh Sandpiper, Grey headed Lapwing |
| III | 0 to 10 | Green Sandpiper, Wood Sandpiper, Common Sandpiper, Red wattled Lapwing, Common Snipe |
| IV | 5 to 12 | Black-winged Stilt |
| V | 10 to 11 | Common Greenshank |
